# Selection for the compactness of highly expressed genes in *Gallus gallus*

**DOI:** 10.1186/1745-6150-5-35

**Published:** 2010-05-14

**Authors:** You S Rao, Zhang F Wang, Xue W Chai, Guo Z Wu, Ming Zhou, Qing H Nie, Xi Q Zhang

**Affiliations:** 1 Department of Biological Technology, Jiangxi Educational Institute, Nanchang, Jiangxi, China; 2 Department of Animal Genetics, Breeding and Reproduction, College of Animal Science, South China Agricultural University, Guangzhou, Guangdong, China

## Abstract

**Background:**

Coding sequence (CDS) length, gene size, and intron length vary within a genome and among genomes. Previous studies in diverse organisms, including human, *D. Melanogaster*, *C. elegans*, *S. cerevisiae*, and *Arabidopsis thaliana*, indicated that there are negative relationships between expression level and gene size, CDS length as well as intron length. Different models such as selection for economy model, genomic design model, and mutational bias hypotheses have been proposed to explain such observation. The debate of which model is a superior one to explain the observation has not been settled down. The chicken (*Gallus gallus*) is an important model organism that bridges the evolutionary gap between mammals and other vertebrates. As *D. Melanogaster*, chicken has a larger effective population size, selection for chicken genome is expected to be more effective in increasing protein synthesis efficiency. Therefore, in this study the chicken was used as a model organism to elucidate the interaction between gene features and expression pattern upon selection pressure.

**Results:**

Based on different technologies, we gathered expression data for nuclear protein coding, single-splicing genes from *Gallus gallus *genome and compared them with gene parameters. We found that gene size, CDS length, first intron length, average intron length, and total intron length are negatively correlated with expression level and expression breadth significantly. The tissue specificity is positively correlated with the first intron length but negatively correlated with the average intron length, and not correlated with the CDS length and protein domain numbers. Comparison analyses showed that ubiquitously expressed genes and narrowly expressed genes with the similar expression levels do not differ in compactness. Our data provided evidence that the genomic design model can not, at least in part, explain our observations. We grouped all somatic-tissue-specific genes (n = 1105), and compared the first intron length and the average intron length between highly expressed genes (top 5% expressed genes) and weakly expressed genes (bottom 5% expressed genes). We found that the first intron length and the average intron length in highly expressed genes are not different from that in weakly expressed genes. We also made a comparison between ubiquitously expressed genes and narrowly expressed somatic genes with similar expression levels. Our data demonstrated that ubiquitously expressed genes are less compact than narrowly expressed genes with the similar expression levels. Obviously, these observations can not be explained by mutational bias hypotheses either. We also found that the significant trend between genes' compactness and expression level could not be affected by local mutational biases. We argued that the selection of economy model is most likely one to explain the relationship between gene expression and gene characteristics in chicken genome.

**Conclusion:**

Natural selection appears to favor the compactness of highly expressed genes in chicken genome. This observation can be explained by the selection of economy model.

**Reviewers:**

This article was reviewed by Dr. Gavin Huttley, Dr. Liran Carmel (nominated by Dr. Eugene V. Koonin) and Dr. Araxi Urrutia (nominated by Dr. Laurence D. Hurst).

## Background

Gene characteristics such as gene size, coding sequence (CDS) length, intron density and intron length, exon density and exon length, as well as amino acid composition are determined by many factors. If natural selection acts on gene sequences, it should favor the sequence variation that makes protein synthesis more efficient or that reduces its cost. Using data on the expression of genes that encode proteins in *Caenorhabditis elegans *and *Homo sapiens*, Castillo-Davis *et al*. [1] demonstrated that introns in highly expressed genes are substantially shorter than those in genes that are expressed at low levels. They argued that natural selection appears to favor shorter introns in highly expressed genes to minimize the cost of transcription and other molecular processes, such as splicing. Urrutia *et al*. [2] gathered expression data for > 10,000 human genes from public datasets obtained by different technologies (SAGE and high-density oligonucleotide chip arrays) and compared them with gene parameters. They found that, even after controlling for regional effects, highly expressed genes coding the smaller proteins, have less intronic DNA and higher codon and amino acid biases. They argued that human genes show signatures consistent with selection mediated by expression level. In *Drosophila*, Lemos *et al*. [3] demonstrated that proteins with higher rates of amino acid substitutions tend to have larger size and tend to be expressed at lower mRNA level, whereas genes with higher levels of gene expression divergence and polymorphism tend to have shorter size and tend to be expressed at higher mRNA level. A negative correlation between evolutionary rate at the protein level and intron size in *Drosophila *was also found by Marais *et al*. [4]. They argued that this correlation is more likely to be explained by the higher abundance of cis -regulatory elements in introns (especially first introns) in genes under strong selective constraints.

Different models have been proposed to explain the relationship between gene features and gene expression pattern in human and other eukaryotes. The selection for economy model [2,5], based on the fact that transcription and translation are slow and expensive processes, argues that natural selection favors the compactness of highly expressed genes. Another distinguished model is the genomic design model [6,7], which suggests that the length of genomic elements is mostly determined by their functional load. In particular, the greater amount of intra- and intergenic noncoding DNA may be involved in the more complex regulation and chromatin-mediated suppression of these genes, whereas the greater length of coding sequences is likely to be related to more complex protein functional domains. Since the observation that highly expressed genes are compact also can be generated by the transcription-associated non-adaptive deletion bias, the third hypothesis be called mutational bias hypothesis has been proposed, which suggests that highly expressed genes tend to localize in chromosomal regions with high deletion rates, or that there is a transcription-associated deletion bias[2,8].

The chicken (*Gallus gallus*) is an important model organism that bridges the evolutionary gap between mammals and other vertebrates. The chicken genome is less than half the size of that of mouse or human, which contains about 20,000-23,000 genes [9]. Chicken karyotype comprises 39 pairs of chromosomes, which are divided into eight pairs of cytologically distinct chromosomes 1-8 (macro-chromosomes) along with Z and W sex chromosomes, and 30 pairs of micro-chromosomes [10]. Micro-chromosomes displayed high recombination rate, high gene density and high G+C content (mean recombination rate, 6.8552 cM/Mb; average gene density, 23.6465 genes/Mb; G+C content, 44.5064%), which distinguished from macro-chromosomes (mean recombination rate, 3.1826 cM/Mb; average gene density, 12.1210 genes/Mb; G+C content, 39.8328%) significantly. A significantly negative correlation between intron length and recombination rate was also found in chicken genome, which implies that longer introns preferred to accumulate in regions of reduced recombination [9]. However, the correlation between the gene characteristics including gene size, CDS length, as well as intron length and the gene expression pattern is not clearly elucidated. With its larger effective population size [9,11,12], selection for chicken genome is expected to be more effective in increasing protein synthesis efficiency. So in this study the chicken was used as a model organism to elucidate the interaction between gene features and expression pattern upon selection pressure. We found that gene size, CDS length, first intron length, average intron length, and total intron length are negatively correlated with expression level and expression breadth. Regression analysis further showed that first intron length is positively correlated with tissue specificity, while the average intron length did not show such correlation. This study will benefit our understanding of how natural selection impacts the gene characteristics in regards of gene expression.

## Results

### Gene size, CDS length, first intron length, average intron length and total intron length are negatively correlated with expression level

**Figure 1:**
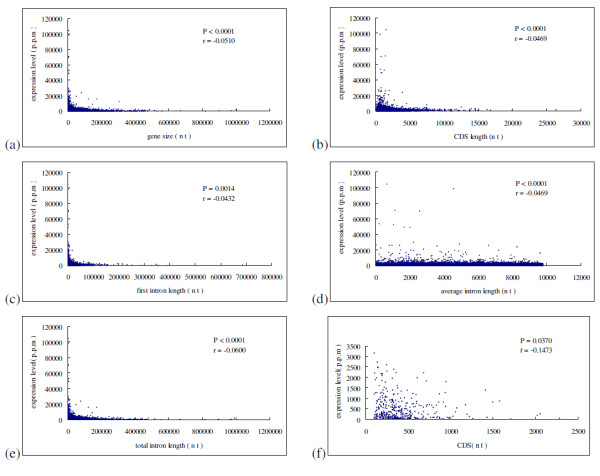
**Gene parameters as functions of expression level**. (a) gene size versus expression level; (b) CDS length versus expression level; (c) first intron length versus expression level; (d) average intron length versus expression level; (e) total intron length versus expression level; (f) CDS length versus expression level for 337 absent intron genes. After adjusted values of type I error using a Bonferroni correction for multiple tests, *P*-values remain statistically significant.

**Figure 2:**
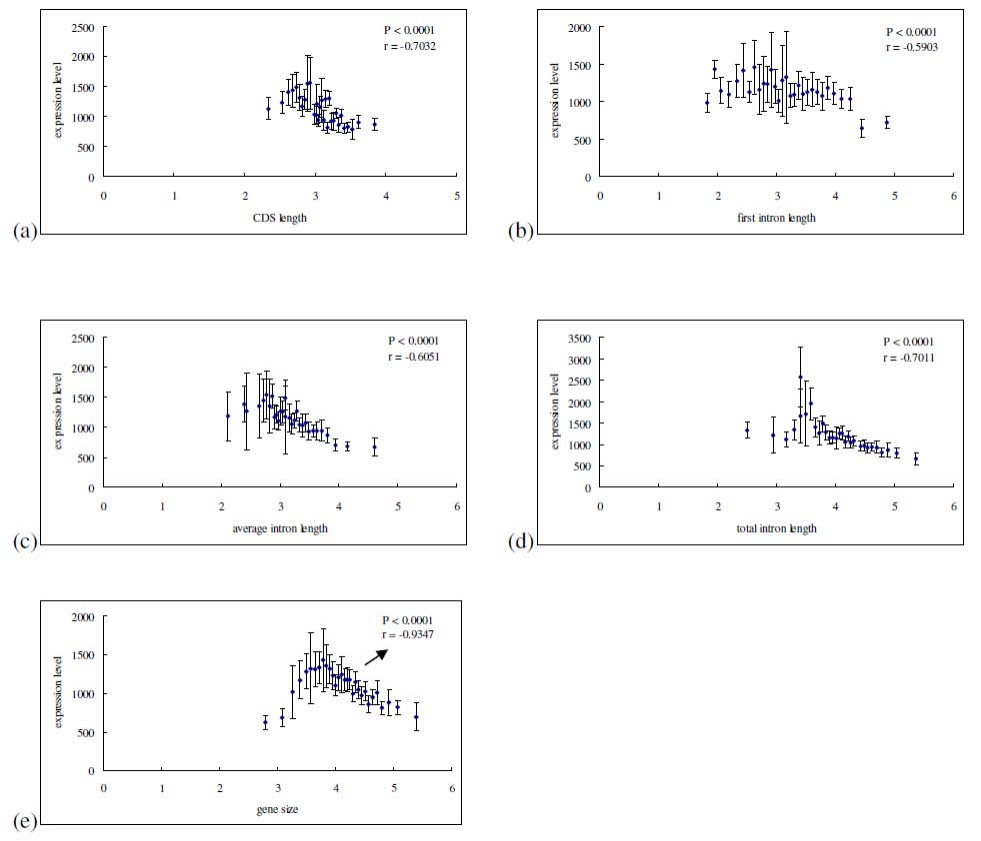
**Average lengths of gene parameters as functions of average expression level category**. Total 10,289 genes grouped into 30 categories based on the length parameters, and then the average lengths (transformed to denary logarithm) and average expression levels (p.p.m) for each category were computed. (a) CDS length versus expression level; (b) first intron length versus expression level; (c) average intron length versus expression level; (d) total intron length versus expression level; (e) gene size versus expression level. The relationship between expression level and gene size shows a "Λ-shape". For the decreasing part, gene compactness shows a strong correlation with gene expression level with very high coefficient (*P *< 0.0001, r = -0.9347). After adjusted values of type I error using a Bonferroni correction for multiple tests, all *P*-values remain statistically significant. Error bars show ± twice the standard error.

Only genes with no evidence of multiple-splicing forms and genes with no obvious annotation errors were included in this study. Genes with introns shorter than 20 bp were discarded. Total number of genes included is10, 289. Linear regression analyses demonstrated that gene size, CDS length, first intron length, average intron length and total intron length are negatively correlated with expression level significantly (see Figure 1a-e). Although nearly all of gene parameters included in the analyses significantly correlated with expression level, most of the associations were weak. In other words, although most of the correlations have low *P *values, they also have low correlation coefficients. It is most likely owing to the fact that the relationship between expression level and gene length parameters is of actually nonlinear [13-15]. According to Urrutia et al. [2], we grouped all genes into 30 categories based on the length parameters, then computed the average length (transformed to denary logarithm) and average expression level for each category. Regression analyses demonstrated that expression level significantly correlated with CDS length, intron length, first intron length, and total intron length with higher coefficient. Over 30% of variation in gene compactness can be explained by gene expression level (see Figure 2a-d). In result similar to Carmel et al. [13], however, the relationship between expression level and gene size shows a "Λ-shape"(see Figure 2e). We employed a segmented regression using the SegReg software, the "Λ-shape" was statistically significant. For the decreasing part, gene compactness shows a strong correlation with gene expression level with very high coefficient (*P *< 0.0001, r = -0.9347). To determine what combination of genes' parameters best predicts expression level, we performed multiple linear regression analyses. The best combination of variables were CDS length and total intron length (*P *< 0.0001, r = -0.6238). Stepwise selection model analyses indicted that CDS length is the most important factor responsible for expression level variation (*P *< 0.0001, r = - 0.7301). For 377 intron-absent genes, we also found a significant trend between expression level and CDS length (see Figure 1f), while such relationship does not exist between gene size and expression level (*P *= 0.8460, r = -0.01003).

### Gene size, CDS length, first intron length, average intron length and total intron length are negatively correlated with expression breadth with high regression coefficient

**Figure 3:**
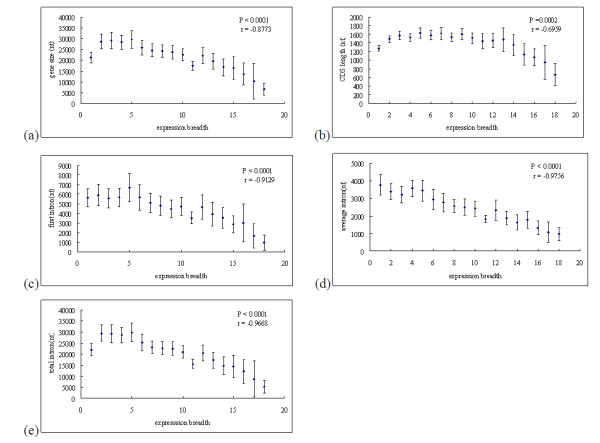
**Scatter plots of average gene size, CDS length, first intron length, average intron length, and total intron length versus gene expression breadth**. (a) gene size versus expression breadth; (b) CDS length versus expression breadth; (c) first intron length versus expression breadth; (d) average intron length versus expression breadth; (e) total intron length versus expression breadth. After adjusted values of type I error using a Bonferroni correction for multiple tests, *P*-values remain statistically significant. Error bars show ± twice the standard error.

Total 10, 289 genes were classified into 18 groups according to their different expression breadth. We then calculated the average gene size, average CDS length, average first intron length, average intron length, and average total intron length. Regression analyses demonstrated that gene sizes, CDS length, first intron length, average intron length, and total intron length showed significantly negative correlation with expression breadth with high regression coefficient (see Figure 3a-e). To determine what combination of genes' parameters best predicts expression breadth, we performed multiple linear regression analyses. The best combination of variables were average intron length and first intron length (*P *< 0.0001, r = - 0.9238). Stepwise selection model analyses indicted that average intron length is the most important factor responsible for breadth variation (*P *< 0.0001, r = - 0.9756).

### The tissue specificity is positively correlated with the first intron length, negatively with the average intron length, but is not correlated with the CDS length and protein domain numbers

**Figure 4:**
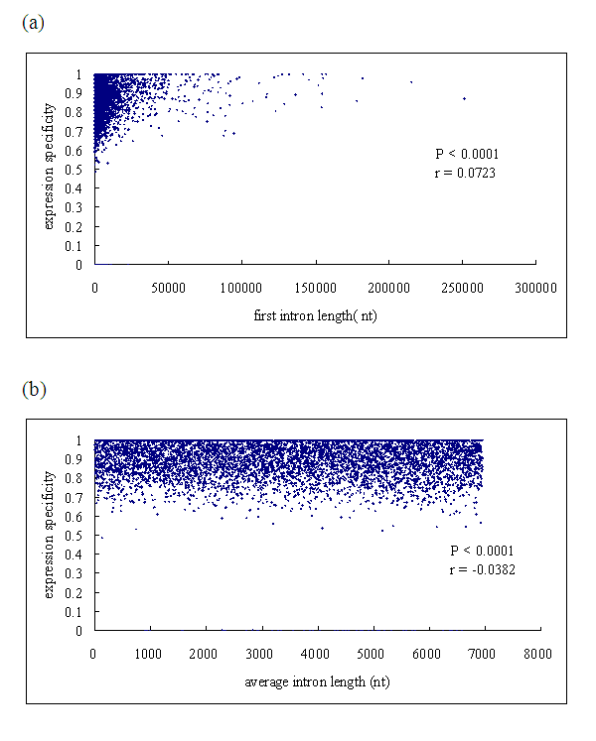
**Scatter plots of the tissue specificity in genes versus first intron length and average intron lengt**. (a) tissue specificity versus first intron length; (b) tissue specificity versus average intron length. After adjusted values of type I error using a Bonferroni correction for multiple tests, *P*-values remain statistically significant.

The tissue specificity index τ measures both qualitative variations (i.e. presence/absence) and quantitative variations of expression level among tissues [16]. Obviously, τ is more representative than expression breadth for the expression complexity of a gene. We made a regression analyses between the tissue specificity and first intron length, average intron length, CDS length, and protein domain numbers. We found that tissue specificity is positively correlated with first intron length (Figure 4a), but negatively correlated with the average intron length (Figure 4b), and is not correlated with CDS length (*P *= 0.0747) and protein domain numbers (*P *= 0.2301).

The genome design hypothesis predicted that the genes with intermediate expression breadths may have higher functional and regulatory complexity, and have larger intron length and protein length [17]. In order to verify this hypothesis, we compared the tissue specificity between the ubiquitously expressed genes (expression breadth ≥ 14) and the intermediately expressed genes (expression breadth = 8 or 9) with similar expression level. Surely, we found that the tissue specificity of intermediately expressed genes is significantly higher than that of ubiquitously expressed genes (τ_intermediate _= 0.8581 ± 0.0059, τ_ubiquitous _= 0.7642 ± 0.0063; t - test, *P *< 0.0001). However, we also found that intermediately expressed genes are not less compact than ubiquitously expressed genes with similar expression levels (CDS length, *P *= 0.3847; average intron length, *P *= 0.2358).

### Genes transcribed in germline cells are not compact

The observation that highly expressed genes are compact also can be generated by the transcription-associated non-adaptive deletion bias. Since only the transcription-associated mutations that occurred in germline cells can be passed on to the organism's offspring, we can suppose that the evolution of somatic-tissue-specific genes (only expressed in one somatic tissue) is free from transcription-associated mutation bias. We grouped all somatic tissue specific genes (n = 1105), and compared the first intron length and average intron length between highly expressed genes (5% top expressed genes) and weakly expressed genes (5% bottom expressed genes). We found that the first intron length and average intron length in highly expressed genes are not different from that in weakly expressed genes (*P *= 0.2031; *P *= 0.4094, respectively), which is consistent with the prediction that the compactness of somatic tissue specific genes are not under transcription-associated mutation bias.

Ubiquitously expressed genes have much higher germline expression level than narrowly expressed somatic genes with the consideration of ectopical expression[18]. If the mutational bias model is applicable to chicken genome, we can expect that the ubiquitously expressed genes have to be more compact than narrowly expressed somatic genes with similar expression levels. Our data demonstrated that ubiquitously expressed genes (the average first intron length = 2980.066; the average intron length = 1654.2) are slightly less compact than narrowly expressed genes (the average first intron length = 2286.601; the average intron length = 1517.867) with similar expression levels, and no significant difference was found (*P *= 0.2213; *P *= 0.5259, respectively).

## Discussion

The relationship between compactness of genes and their expression pattern is one of the signs how natural selection may impact the evolution of genomes. Previous studies in diverse organisms including *S. Cerevisiae*, *C. Elegans*, *D. Melanogaster*, *Arabidopsis thaliana*, and humans have showed that natural selection appears to favor the compactness of highly expressed genes [1-7,19,20]. In this study, we found that gene size, CDS length, first intron length, average intron length and total intron length are negatively correlated with expression level and expression breadth significantly. Our study demonstrated that like in other genomes, natural selection also favors highly expressed genes at higher compactness in chicken genome.

Introns are widespread and abundant in eukaryotic genomes, which impose a burden on organisms harboring them in terms of the energy, time, and materials required for both DNA replication and gene transcription. Many studies indicated that intron size varies within the genome and among genomes, and is influenced by various factors [21-26]. In order to explain the relationship between gene characteristics and gene expression, different models, such as selection for economy model, genomic design model and mutational bias hypotheses have been proposed. Recently, some studies in Drosophila and human showed that regulatory elements often enriched in introns, especially in the first introns. This seems to provide some evidence for the genome design model [27-31]. Vinogradov investigated the consecutive local alignments between human and mouse intronic DNA sequences, He found that the introns of tissue-specific genes tend to have higher fraction of aligned sequences than those of house-keeping genes, the amount of aligned intronic DNA correlates with the number of protein domains significantly [6]. In the present study, we observed that the connection between gene expression breadth in chicken and gene compactness to be significantly stronger than the connection between expression level and compactness, and the tissue specificity of genes is positively correlated with first intron length (*P *< 0.0001, r = 0.07276). This seems to be compatible with the genome design hypothesis. However, we also found the tissue specificity negatively correlated with average intron length (*P *< 0.0001, r = -0.03823), and not correlated with CDS length (*P *= 0.0963) and protein domain numbers (*P *= 0.2301). To test whether functional complexity of tissue-specific expression has produced both large introns and large proteins in tissue-specific genes [32], we compared genes with similar expression levels but differing greatly in expression breadth (ubiquitously expressed genes and narrowly expressed genes with similar expression levels). Comparison analyses showed that ubiquitously expressed genes and narrowly expressed genes with the similar expression levels do not differ in compactness (*P *= 0.2386). We also found that intermediately expressed genes are not less compact than ubiquitously expressed genes with similar expression levels (CDS length, *P *= 0.3847; average intron length, *P *= 0.2358). This implies that the genome design hypothesis can not, at least in part, explain our observations.

The observation that highly expressed genes are compact can also be explained by the hypothesis of transcription-associated non-adaptive deletion bias, because introns may be lost through a recombination between a reverse transcript and the corresponding genomic DNA, and highly expressed genes have more potential substrates (i.e. mRNA) for reverse transcription, and thus are more likely to lose their introns [33-35]. To test whether this model is applicable to chicken genome, we grouped all somatic-tissue-specific genes (n = 1105), and compared the first intron length and average intron length between highly expressed genes (5% top expressed genes) and weakly expressed genes (5% bottom expressed genes). We found that the first intron length and average intron length in highly expressed genes are not different from those in weakly expressed genes (*P *= 0.2031; *P *= 0.4094 respectively). We also compared ubiquitously expressed genes and narrowly expressed somatic genes with similar expression levels. Our data demonstrated that ubiquitously expressed genes are slightly less compact than narrowly expressed genes with the similar expression levels, and no significant difference was found. Obviously, this model can not be supported by our data either.

Li *et al*. investigated the relationship between gene expression pattern and genes features for human, *M. musculus*, and *A. thaliana*. They found evidences that genes with high functional/regulatory complexity did not have longer introns and longer proteins. They also found that house-keeping genes were not more compact than the narrowly expressed somatic genes with similar average expression levels. They concluded that their findings support the selection of economy model [19]. Carmel *et al*. carried out an analysis of gene architecture and expression levels of four organisms, *Homo sapiens*, *Caenorhabiditis elegans*, *Drosophila melanogaster*, and *Arabidopsis thaliana*, revealed a surprising, nonmonotonic, universal relationship between expression level and gene compactness. They found that the connection between gene expression breadth in humans and gene compactness to be significantly weaker than the connection between expression level and compactness, which is compatible with the selection hypothesis but not the genome design hypothesis [13]. Using a collection of expressed sequence tag (EST) data, Zhu *et al*. investigated the breadth of expression for 17,288 human RefSeq loci across 18 human tissues. They found that the negative correlation between expression level and length parameters is actually nonlinear. Highly expressed genes are rarely large, however, poorly expressed genes show a broad range of variations in gene length. They argued that the selection on gene length for economy might only act on the larger genes [14]. In the present study, we found that gene length parameters in highly expressed genes are correlated with genes' compactness positively. To determine whether our findings could be affected by local mutational biases [2], we computed average G+C content for all introns and cDNA. The G+C content did not show any significant trend with expression level (*P *= 0.2768; *P *= 0.1519, respectively). We also made an analysis between recombination rate and average expression level of genes. No significant trend was found, too (*P *= 0.6589). Especially, for 377 intron-absent genes, we found that CDS length are negatively correlated with expression level significantly (*P *= 0.0370, r = -0.1073). This means that the selection of economy model is the most likely hypothesis to explain the relationship between genes expression pattern and genes characteristics in chicken genome.

The selection of economy model falls into two conflictive hypotheses, the energetic cost hypothesis and the time cost hypothesis. The first argues that selection for compact genes may be driven by minimizing the energetic cost of transcription [1,13,14,16], while the second one states that selection in favor of short intron and short exon is likely due to the requirement to transcribe large amounts of mRNA molecules within limited periods [24,25,35,36]. The negative correlation between gene size, CDS length and intron length with expression level (including 377 intron-absent genes) in chicken genome seems to provide some evidence for the energetic cost hypothesis. Otherwise, regression analysis between intron number and expression level in this study demonstrated that intron number inversely correlated with expression level at 10% significant level (*P *= 0.0895, r = -0.0201; see Additional file 1). Since splicing more introns requires more time than transcription and becomes ratelimiting [24], this seems to be consistent with the time cost hypothesis. However, Singh et al. suggested that splicing must occur co-transcriptionally, irrespective of the class of introns and intron length, because the splicing reaction is completed well before the gene is transcribed to the end [37]. In fact, the energy cost is closely correlated with the time cost in the process of transcription, splicing and translation. Transcribing a larger gene also requires more time expenditure and more energy cost. Meanwhile, removal of more introns from a pre-mRNA also incurs more energy and time cost. Although no energy required in the transesterificiation reaction, the spliceosome formation and mRNA conformation changes need hydrolysis of ATP to energize. Probably, the effect of the natural selection is to insure the function of the gene, and optimize the expenditure for time, energy, materials, and others.

Similar to *H. sapiens*, *D. melanogaster *and *A. thaliana *[13,38], we also found that intron density (intron number per Kb CDS) shows a positive correlation with expression level (*P *< 0.0001, r = 0.07516; see Additional file 1). Carmel et al. argued this positive correlation is compatible with their findings that the initial gene elongation, in particular, in introns, would increase gene' expression level [13]. Our data did show the similar trend (see Figure 2e). Perhaps, the positive relationship between intron density and expression level is likely to suggest that introns may play an important role in the process of gene expression in eukaryotic cells, and reflects that other factors are working to increase the density of introns in highly expressed genes.

## Conclusion

Natural selection appears to favor the compactness of highly expressed genes in chicken genome. This observation can be explained by the selection of economy model.

## Materials and methods

### Genome annotation

Genes' parameters were taken from Genomon gene models from the NCBI ftp://ftp.ncbi.nih.gov/genomes/) chicken map viewer Web site (Build 2.1, released November, 2006) that are displayed as Genes OnSequence.

Only nuclear genes with complete information on protein-coding sequence, and no evidence of multiple-splicing forms were included. The products of genes can be found from http://www.ncbi.nlm.nih.gov/UniGene/. Some genes with a partial protein-coding sequence (CDS) were not included. Many genes have multiple mRNA (multiple splicing) also ruled out. Some genes' CDS length is not consistent with the total length of all exons of gene obviously. These genes were defined annotation errors and were not included in this study, too. Finally, total 10, 289 genes' parameters, including gene size, CDS length, first intron length, average intron length, and total intron length were estimated. Gene compactness was defined as genes with shorter size, shorter intron length, or shorter CDS length.

### Expression datasets

Two gene expression datasets were included in this study. Gene expression dataset 1, EST databases, were taken from the NCBI FTP site. 633,321 EST sequences were available. We used the number of EST sequences in this databases that align unequivocally to a given gene, and compared the set of chicken mRNA/cDNA sequences with the ESTs using the program BLASTN. We accepted EST hits of > 400 nt and with > 96% identity to a mRNA/cDNA sequence as matches. If they showed > 98% identity, we accepted hits of 100 - 400 nt, and we discarded hits of < 100 nt [1]. After excluding genes with multiple-splicing forms and genes with obvious annotation errors, the data on 10, 289 genes for 18 tissues were taken into account: blood, brain, cecum, connective tissue, embryonic tissue, epiphyseal growth plate, gonad, head, heart, limb, liver, muscle, ovary, pancreas, spleen, testis, and thymus. Tags per million were then calculated for each tissue of each gene. Two measures of expression level were defined: total expression level, which is the sum of the total 18 tissues' EST, and expression breadth, the numbers of tissues in which EST was found. EST-based method was used to identify genes' expression breadth [14]. Genes were defined as ubiquitously or narrowly expressed if they are expressed in > 14 tissues, or < 3 tissues, respectively (when "> 15 or < 2", "> 16 or < 2" defined, we get the similar result). For somatic cells, narrowly expressed genes were defined as those expressed in less than 20% of total normal samples excluding germline cells, reproductive organs, or early developmental stage. The tissue specificity index (τ) measured both qualitative variations (i.e. presence/absence) and quantitative variations of expression level among tissues, was defined as:

Where *N *is the number of tissue samples examined, *x_i _*is the expression level of the gene in sample *i*, and *x_max _*is the highest expression level of the gene across the *N *samples examined [16]. The protein characters were estimated using the SwissPfam version 20 http://pfam.janelia.org/.

Gene expression dataset 2 derived from a high-density oligonucleotide chip arrays, GSE12974 (GEO, http://www.ncbi.nlm.nih.gov/geo) [39]. As recommended [40], a gene was assumed to be expressed in a tissue significantly if its intensity exceeded the 99th percentile of intensities from the negative controls (Using 90% and 95% as the thresholds gave similar results, date not show). After excluding genes with multiple-splicing forms and genes with obvious annotation errors, only 4, 086 genes for 20 tissues were taken into account: Bursa of fabricius, cerebellum, cerebral cortex, eye, femur with bone marrow, gallbladder, gizzard, heart, intestine, kidney, liver, lung, muscle, ovary, oviduct, skin, spleen, stomach, testis and thymus. As the two expression datasets given the similar result, we only displayed the result of dataset 1 in this report (The result of dataset 2 can be seen from Additional files 2, 3).

### Recombination rate estimate

The recombination rates for 4 Mb windows were estimated. The versions of the genome assemblies (NCBI build 2.1, released November, 2006) and genetic linkage map WUR (NCBI Mapview build 2.1) were used. Locations of individual markers were determined based on alignments of the full sequence of the marker using BLAST. Markers placement information is available for download from the UCSC Genome Browser (Kent *et al*. 2002, http://www.genome.ucsc.edu). The linear function was fit to the points representing genetic and physical map position in 4 Mb windows. The slope of this line was taken as the estimate of recombination rate [41]. When only two markers were anchored to the sequence, a straight line was calculated [42]. Total windows included is 210, covering approximately 80% of the chicken genome. Eleven windows contain only two markers. The average expression level for each window was estimated based on genes' expression level located in this window.

## Competing interests

The authors declare that they have no competing interests.

## Authors' contributions

YSR, XQZ and QHN conceived and designed the experiments. ZFW and GZW analyzed the data. XWC and MZ collected the expression data. YSR and XQZ wrote the paper. All authors read and approved the final manuscript.

## Reviewer's report 1

### Gavin Huttley, John Curtin School of Medical Research, Australian National University

1. This article addresses a very interesting question concerning the structure of genes and the nature of gene expression. In eukaryotes, previous work has demonstrated a number of significant such correlations. These include a negative correlation between intron length and: recombination rate (Carvalho and Clark. Nature 401: 344); the rate of protein sequence evolution (Marais et al Genetics 170: 481-5); and level of expression (Castillo-Davis et al Nature Genetics 31: 415-8). A number of hypotheses have been proposed to account for these correlations and the manuscript by Rao et al seeks to distinguish between them through analyses of the chicken genome.

While the authors conduct some interesting analyses the work suffers from a number of flaws that weaken the inferences drawn. These include errors in language, an incomplete description of the methods and over interpretation of results.

While large volumes of genome sequence data can provide enormous statistical power they also present challenges. As illustrated in this study, many hypothesis tests that use genomics data prove strikingly significant. For instance, in Figures 1 multiple probabilities of association < 10^-4 ^are reported. Such very small probabilities are common in analyses of such large data sets and thus the significance of a hypothesis test is unfortunately common. A more meaningful perspective on the analyses can be obtained by considering the percentage of variance in the data correctly predicted by the correlation. In the current case, if we take the very optimistic view that all reported analyses in Figures 1 are statistically independent (more on this below) the results are much less striking. The summed R^2 ^for the correlation coefficients reported in the figures is only 3% and if we remove nominally significant results (as these would likely be discarded if multiple test corrections were actually applied) the R^2 ^reduces to <1%. This means that 99% of the variation in gene compactness is not explained by the gene expression levels. From this perspective, the authors interpretation that the compactness of a gene derives from natural selection operating via the "economy model" on gene expression is very poorly supported.

**Author's response**: Surely, the gene expression level shows a strong significant trend with gene size, CDS length, and intron length, but with lower coefficient. It is most likely owing to the fact that the correlation between expression level and length parameters is of actually nonlinear (Zhu et al. 2008; Carmel et al. 2009; Yang et al. 2009). According to Urrutia et al. (2003), we reanalyzed our date sets (see Figure 2). We grouped all genes into 30 categories based on the length parameters, then computed the average length (transformed to denary logarithm) and expression level for each category. Regression analyses demonstrated that expression level significantly correlated with average intron length, first intron length, total intron length and CDS length with higher coefficient. Approximate 30-40% of variation in gene compactness can be explained by gene expression level. In result similar to Carmel et al. (2009), however, the relationship between expression level and gene size shows a "Λ-shape". We employed a segmented regression using the SegReg software, and the "Λ-shape" was statistically significant. For the decreasing part, gene compactness shows a strong correlation with gene expression level with very high coefficient (*P *< 0.0001, r = -0.9347). To determine what combination of genes' parameters best predicts expression level, we performed multiple linear regression analyses. The best combination of variables were CDS length and total intron length (*P *< 0.0001, r = -0.6238). Stepwise selection model analyses indicted that CDS length is the most important factor responsible for expression level variation (*P *< 0.0001, r = - 0.7301). These results have been integrated to the revised manuscript.

2. A further difficulty with this work is the failure to adhere to statistical standards in defining "significance" including absence of any corrections for testing so many hypotheses. For instance, in the initial draft of the manuscript the authors suggested that "ubiquitously expressed genes are less compact than narrowly expressed genes with similar expression levels (*P *= 0.2386)". A conventional interpretation of this probability would be that these gene expression classes do not differ in compactness. Further compounding such errors in interpretation of hypothesis tests is that all such tests are treated as independent with no correction for multiple testing applied.

**Author's response**: After adjusted values of type I error using a Bonferroni correction for multiple tests, *P*-values remain statistically significant for Figure 1a-e, Figure 2a-d, Figure 3a-e, Figure 4a-b. For Additional file 2, the adjusted *P *-values for Figure 2a and Figure 2c remain statistically significant, and the adjusted *P*- value for Figure 2b and Figure 2d were 0.371 and 0.204, respectively. For Additional file 3, the adjusted *P*- value for Figure 3b-d remains statistically significant, and the adjusted *P*- value for Figure 3a is 0.122.

In order to test whether functional complexity of tissue-specific expression has produced both large introns and large proteins in tissue-specific genes, we compared genes' intron length with similar expression levels but differing greatly in expression breadth (ubiquitously expressed genes and narrowly expressed genes with similar expression levels). We found no significant difference between them. The sentence "Comparison analyses showed that ubiquitously expressed genes are less compact than narrowly expressed genes with similar expression levels (*P *= 0.2386)" has been corrected to "Comparison analyses showed that ubiquitously expressed genes and narrowly expressed genes with the similar expression levels do not differ in compactness (*P *= 0.2386)".

Yet another complication affecting the interpretation is that many of their metrics are not statistically independent of each other. For instance, the metrics of gene size, first intron length and average intron length are clearly related and cannot be considered independent. As a result the probabilities from such tests are expected to be related.

**Author's response**: Surely, gene's metrics are not statistically independent of each other. We found that gene size is positively correlated with CDS length, and average intron length significantly (*P *< 0.0001). Average intron length also shows a significant trend with first intron length (*P *< 0.0001). After adjusted by CDS length and average intron length, the gene size still shows significant trend with expression level, respectively (*P *< 0.0001, r = -0.05; *P *< 0.0001, r = -0.047). After adjusted by first intron length, the average intron length also shows a significant trend with expression level (*P *= 0.0015, r = -0.045).

4. The principal conclusion to be drawn from this work is that what process(es) cause gene compactness remain an open question.

**Author's response**: According to Urrutia et al. (2003), we reanalyzed our datesets (see Figure 2a-e). We found that gene expression level significantly correlated with intron length, first intron length, total intron length and CDS length with higher coefficient. Approximate 30-40% of variation in gene compactness can be explained by gene expression level. We also found that gene size, CDS length, first intron length, average intron length, and total intron length showed significantly negative correlation with expression breadth with high regression coefficient (see Figure 3a-e). Therefore, we argued the principal conclusion drawn from this work is believable.

## Reviewer's report 2

### Liran Carmel, National Center for Biotechnology Information, National Library of Medicine, National Institutes of Health, Bethesda, MD 20894, USA

1. The relationship between gene compactness and expression was investigated considerably during the recent years. Chicken, as far as I know, was not included so far in those analyses and therefore this manuscript is a welcome addition. In general, the analysis is solid and the paper is well written, but I do have several comments. In the Methods section, the authors do not describe the gene features they use in sufficient detail. For example, I'm not sure what is the exact difference between peptide length and CDS length.

**Author's response**: CDS refers to the complete coding sequence of a gene. CDS corresponding to all annotated genes in the chicken genome were downloaded from http://www.ncbi.nlm.nih.gov/sites/gquery. Peptide information for genes derived from http://www.ncbi.nlm.nih.gov/protein. As genes with multiple-splicing forms, incomplete CDS sequences, and obvious annotation errors were excluded from this study, we checked up our raw data again and found that CDS length is three times of peptide length plus 3 nt. Since the CDS length highly correlated with peptide length, the peptide length parameter was discarded. The following additional details on methods have been integrated into the revised manuscript.

Genes' parameters were taken from Genomon gene models from the NCBI chicken map viewer Web site (Build 2.1) that are displayed as Genes OnSequence. The products of genes can be found from http://www.ncbi.nlm.nih.gov/UniGene/. Some genes with a partial protein-coding sequence (CDS) were not included in this study. Many genes have multiple mRNA (multiple splicing) also ruled out. Some genes' CDS length is not consistent with the total length of all exons of gene obviously. These genes were defined annotation errors and were not included, too.

Genes were defined as ubiquitously or narrowly expressed if they are expressed in > 14 tissues, or < 3 tissues, respectively (when "> 15 or < 2", "> 16 or < 2" defined, we get the similar result).

In this study, the recombination rates for 4 Mb windows were estimated. Total windows included is 210, covering approximately 80% of the chicken genome. Eleven windows contain only two markers.

2. In the Results section, I'm concerned about the value of the correlation coefficients. It is true that many of them are statistically significant, but is there really a meaning to correlation coefficients that are well below 0.1 (most parts of Figure 1)? This means that the explained variance is lower than 1%! Part of the problem seems to be in nonlinear relationships between the variables. Perhaps using Spearman correlation can improve things.

**Author's response**: According to Urrutia et al. (2003), we reanalyzed our datasets (see Figure 2). We found that genes expression level significantly correlated with intron length, first intron length, total intron length and CDS length with higher coefficient. Approximate 30-40% of variation in gene compactness can be explained by gene expression level.

3. The authors look at the first intron length, and at the average intron length. I think that in the context of the study, total intron length is more relevant than average intron length. I believe the authors should add this feature to their list of features.

**Author's response**: This feature has been integrated to the revised manuscript (see Figure 1e, Figure 2e, Figure 3e).

4. In the Discussion, the authors claim that the negative correlation between gene size, CDS length and peptide length with expression level provides some evidence for the energetic cost hypothesis. I'm not sure that I understand why. These correlations were well known at the time that the genomic design hypothesis was suggested.

**Author's response**: The selection for economy model believes that natural selection favors for short gene size, short proteins (short CDS) and short introns to minimize the energetic and time cost of gene expression, as gene's transcription and translation are slow and expensive processes. In contrast, the genome design model suggests that the length of genomic elements is mostly determined by their functional load. In particular, the greater amount of intra- and intergenic noncoding DNA may be involved in the more complex regulation and chromatin-mediated suppression of these genes, whereas the greater length of coding sequences is likely to be related to more complex protein functional domains. Obviously, the debate of which model is a superior one to explain the relationship between gene compactness and expression pattern has not been settled down.

5. The authors claim in the Discussion that a lot of studies support the genomic design model. Actually, I'm under the opposite impression. For example, the following papers provide evidence against the genomic design hypothesis: Eisenberg and Levanon 2003 (PMID: 12850439); Urrutia and Hurst 2003 (PMID: 12975314); Chen et al. 2005 (PMID: 15797613); Seoighe, Gehring, and Hurst 2005 (PMID: 16110339); Li, Feng, and Niu 2007 (PMID: 17610841).

**Author's response**: These sentences were rewritten to "some studies in Drosophila and human showed that regulatory elements often enriched in introns, especially in the first introns. This seems to provide some evidence for the genome design model".

6. The manuscript would benefit from a careful proofreading. Some typos that I found: "This seems that" (top line of page 3), "Many study" (Discussion), "After analyzed" (Discussion).

**Author's response**: It has been corrected.

## Reviewer's report 3

### Araxi Urrutia, Royal Society Dorothy Hodgkin Research Fellow at the Department of Biology and Biochemistry, University of Bath, BA2 7AY, UK

I think this paper offers a comprehensive analysis of the relationship between expression patterns and gene compactness in the chicken genome. Using publicly available EST libraries from several chicken tissues, the authors analyzed the relation between expression intensity and expression breadth with intron and CDS length of genes. Their results support that highly expressed genes but probably not broadly expressed genes (see discrepancies between dataset 1 and 2) and certainly not genes with complex expression patterns have more compact introns and proteins. The authors conclude that their results support that highly expressed genes are under selection to minimize energy and time expenditure in transcription/translation and reject other models previously put forward to explain the relationships between expression patterns and gene compactness.

General comments in no particular order:

1. While the authors describe in some detail previous studies linking expression levels to gene compactness in several organisms. Arabidopsis is left out. In PLoS One. 2009; 4(7): e6356, the authors show that gene expression intensity but not breadth is associated with compact genes. Their findings support that while highly expressed genes are under selection for transcription economy, genes expressed in more tissues are not.

**Author's response**: This paper has been cited in the revised manuscript.

2. The authors repeatedly state that every effort was taken to eliminate genes with alternative splicing variants. I am afraid that this might be a futile endeavour. A recent paper [Nucleic Acids Research, 2007, Vol. 35, No. 1 125-131] showed that after correcting for differences in EST coverage, both human and chicken appear to have very similar levels of alternative splicing. Since deep sequencing analyses have no revealed that up to 9%of human genes undergo alternative splicing, we can assume that a similar percentage of the chicken genes also produce more than one isoform. Given this, I see no reason to make any efforts to eliminate genes with alternative splicing.

**Author's response**: Above study indicated that over 40% of chicken genes, corresponding to 8,000-9,200 genes, undergo alternative splicing. It means approximate 12,000-13,800 genes have a single splicing isoform. Why we only collected genes with complete information on protein-coding sequences, and no evidence of multiple-splicing forms is that we can get a relatively accurate estimate for genes' parameters. Although further studies may reveal more genes undergo alternative splicing, we believe this can not affect our results.

3. Breadth and intensity of expression are highly related, the use of multiple regression analyses could help separate the effects of expression intensity and breadth on gene characteristics.

**Author's response**: Surely, expression breadth and expression intensity are positively correlated (*P *< 0.0001, r = 0.4096). Multiple regression analyses implied that expression breadth is a main factor to shape gene's compactness.

4. While the authors conclude that the lack of large genes with intermediate expression breadth points against the "genomic design" model, the fact that higher tissue specificity is associated with longer first introns but not otherwise bigger subsequent introns or peptide sequences suggests that first intron length is actually being shaped by selection to position regulatory elements in this region and therefore opposing to some extent the effects of the selection for "economy". Once more, multiple regression analyses could probably help to clear the relationships among all these parameters.

**Author's response**: In this study, we observed that the connection between gene expression breadth and gene compactness to be significantly stronger than the connection between expression level and compactness; the tissue specificity of gene is positively correlated with first intron length (*P *< 0.0001, r = 0.07276); multiple regression analyses also showed that expression breadth is a main factor to shape gene's compactness. This seems to be compatible with the genome design hypothesis. However, we also found some evidence against the genome design model: 1) The tissue specificity negatively correlated with average intron length (*P *< 0.0001, r = -0.03823), and not correlated with CDS length (*P *= 0.0963) and protein domain numbers (*P *= 0.2301); 2) Comparison between genes with similar expression levels but differing greatly in expression breadth (ubiquitously expressed genes and narrowly expressed genes with similar expression levels) showed that ubiquitously expressed genes and narrowly expressed genes with the similar expression levels do not differ in compactness (*P *= 0.2386); 3) The intermediately expressed genes are not less compact than ubiquitously expressed genes with similar expression levels (CDS length, *P *= 0.3847; average intron length, *P *= 0.2358). Therefore, we concluded that the genome design hypothesis can not, at least in part, explain our observations.

To determine what combination of genes' parameters best predicts expression breadth, we performed multiple linear regression analyses. The best combination of variables were average intron length and first intron length (*P *< 0.0001, r = - 0.9238). Stepwise selection model analyses indicted that average intron length is the most important factor responsible for breadth variation (*P *< 0.0001, r = - 0.9756). For expression level, the most important factor responsible for expression level variation is CDS length (*P *< 0.0001, r = - 0.7301).

5. No reasons are given as to why microarray and EST datasets differ in their results for breadth of expression. While in EST datasets most genes are "not expressed" in most tissues, with microarray datasets, the number of genes "expressed" per tissue tends to be higher depending on the thresholds chosen. Authors should comment on this.

**Author's response**: Previous studies indicted that genes' expression breadth based on microarray date tend to be underestimated (Zhu, 2008; Zhu and Yu 2008). In the present study, gene expression dataset 2 derived from a high-density oligonucleotide chip arrays, GSE12974 (GEO, http://www.ncbi.nlm.nih.gov/geo) (Chan et al. 2009). As recommended (Zhang et al. 2004), a gene was assumed to be expressed in a tissue significantly if its intensity exceeded the 99th percentile of intensities from the negative controls (Using 90% and 95% as the thresholds gave similar results). In fact, the expression breadth for dataset 2 is correlated with the gene size, CDS length, first intron length, average intron length and total intron length (see Additional file 3), but with lower coefficient compared to dataset 1. The main difference between them is that, after adjusted values of type I error using a Bonferroni correction for multiple tests, *P*-values for dataset 1 remain statistically significant, *P*-value for Fgure 3b-d of dataset 2 (see Additional file 3) remains statistically significant, but that for Figure 3a is not (see Additional file 3).

Specific comments:

1. Add reference for *Genome Res*. 2003 13: 1998-2004 which also shows that gene compactness is related to expression patterns.

**Author's response**: It has been cited.

2. Abstract is too long.

**Author's response**: It has been corrected.

3. Correlation coefficients and p values are not included in many of the analyses but mentioned only on the figures; since coefficients are included when there is no corresponding figure it would be easier to follow the text if all r and p values where there.

**Author's response**: According to the Biology Direct, coefficients and *P*-values were not included in the text after listed on the figures or tables.

4. It would help if the authors stated what each possible result from each analysis would mean in relation to the different models. At the moment interpretation of the results require a lot of familiarity with the topic, so briefly describing the predictions of each model in the introduction or at the beginning of each section in the results would make the paper more accessible to those not specifically working on gene size and expression levels.

**Author's response**: It has been done.

Minor comments:

Overall very readable and fluent manuscript but there are lots of typos and grammatical mistakes.

**Author's response**: The whole paper was revised carefully by a native English speaker.

## Supplementary Material

Additional file 1 **Scatter plots of intron number, intron density versus expression level.** (a) intron number versus expression level (r = -0.02006, *P *= 0.0895); (b) intron density versus expression level (r = 0.07516, *P *< 0.0001). Relationship between intron number and intron density with expression level based on dataset 1. After adjusted values of type I error using a Bonferroni correction for multiple tests, the adjusted *P*- value for (b) remain statistically significant. Click here for file

Additional file 2 **Scatter plots of CDS length, gene size, first intron length, average intron length versus gene expression level.** (a) CDS length versus expression level (r = -0.4128, *P *= 0.01187); (b) gene length length versus expression level (r = -0.3122, *P *= 0.09273); (c) first intorn length versus expression level (r = -0.6679, *P *= 0.00683); (d) average intorn length versus expression level (r = -0.3978, *P *= 0.05106). Gene size, CDS length, first intron length, and average intron length are negatively correlated with expression level based on dataset 2. After adjusted values of type I error using a Bonferroni correction for multiple tests, the adjusted *P*- value for (a)and (c) remain statistically significantly, the adjusted *P*- value for (b) and (d) were 0.371 and 0.204, respectively. Click here for file

Additional file 3 **Scatter plots of average gene size, CDS length, first intron length, average intron length versus gene expression breadth.** (a) gene size versus expression breadth (r = -0.24842, *P *= 0.03051); (b) CDS length versus expression breadth (r = -0.54325, *P *= 0.0133); (c) first intron length versus expression breadth; (r = -0.8081, *P *∠ 0.001); (d) average intron length versus expression breadth(r = -0.80437, *P *∠ 0.001). Error bars show ± twice the standard error. Gene size, CDS length, first intron length and average intron length are negatively correlated with expression breadth based on dataset 2. After adjusted values of type I error using a Bonferroni correction for multiple tests, the adjusted *P*-value for (b) - (d) remaining statistically significant, the adjusted *P*- value for (a) is 0.122. Click here for file

## References

[B1] Castillo-Davis CI Mekhedov SL Hartl DL Koonin EV Kondrashov FA Selection for short introns in highly expressed genes Nat Genet 2002 31 415 418 12134150 10.1038/ng940

[B2] Urrutia AO Hurst LD The signature of selection mediated by expression on human genes Genome Res 2003 13 2260 2264 10.1101/gr.641103 12975314 PMC403694

[B3] Lemos BC Meiklejohn CD Caceres M Hartl DL Rates of divergence in gene expression profiles of primates, mice and flies: stabilizing selection and variability among functional categories Evolution 2005 59 126 137 15792233

[B4] Marais G Nouvellet P Keightley PD Charlesworth B Intron Size and Exon Evolution in *Drosophila* Genetics 2005 170 481 485 10.1534/genetics.104.037333 15781704 PMC1449718

[B5] Seoighe C Gehring C Hurst LD Gametophytic selection in Arabidopsis thaliana supports the selective model of intron length reduction PLoS Genet 2005 1 2 e13 10.1371/journal.pgen.0010013 16110339 PMC1186733

[B6] Vinogradov AE 'Genome design' model: evidence from conserved intronic sequence in human- mouse comparison Genome Res 2006 16 347 354 10.1101/gr.4318206 16461636 PMC1415212

[B7] Eisenberg E Levanon EY Human housekeeping genes are compact Trends Genet 2003 19 7 362 365 10.1016/S0168-9525(03)00140-9 12850439

[B8] Comeron JM Selective and mutational patterns associated with gene expression in humans: Influences on synonymous composition and intron presence Genetics 2004 67 3 1293 1304 10.1534/genetics.104.026351 PMC1470943 15280243

[B9] International Chicken Genome Sequencing Consortium Sequence and comparative analysis of the chicken genome provide unique perspectives on vertebrate evolution Nature 2004 432 695 716 10.1038/nature03154 15592404

[B10] Groenen MA Cheng HH Bumstead N Benkel BF Briles WE Burke T Burt DW Crittenden LB Dodgson J Hillel J Lamont S de Leon AP Soller M Takahashi H Vignal A A consensus linkage map of the chicken genome Genome Res 2000 10 1 137 47 10645958 10.1101/gr.10.1.137 PMC310508

[B11] Rao YS Wang ZF Zhou M Shen X Xia MN Zhang XQ Comparative study of SNP diversity and calculation of the effective size of population in chicken Hereditas (Beijing) 2007 29 9 1083 1088 10.1360/yc-007-1083 17855258

[B12] International chicken polymorphism map consortium A genetic variation map for chicken with 2.8 million single-nucleotide polymorphisms Nature 2004 432 717 725 10.1038/nature03156 15592405 PMC2263125

[B13] Carmel L Koonin E A universal nonmonotonic relationship between genen compactnes and expression level in muticellular eukaryotes Genome Biol Evol 2009 doi:10.1093/gbe/evp038 20333206 10.1093/gbe/evp038 PMC2817431

[B14] Zhu J He FH Hu SN Yu J On the nature of human housekeeping genes Trends in Genet 2008 24 481 484 10.1016/j.tig.2008.08.004 18786740

[B15] Yang HX In plants, expression breadth and expression level distinctly and non-linearly correlate with gene structure Biology Direct 2009 4 45 10.1186/1745-6150-4-45 19930585 PMC2794262

[B16] Yanai I Benjamin H Shmoish M Chalifa-Caspi V Shklar M Ophir A Bar-Even S Horn-Saban M Safran E Domany D Lancet R Shmueli O Genome-wide midrange transcription profiles reveal expression level relationships in human tissue specification Bioinformatics 2005 21 650 659 10.1093/bioinformatics/bti042 15388519

[B17] Vinogradov AE 'Genome design' model and multicellular complexity: golden middle Nucleic Acids Res 2006 34 5906 5914 10.1093/nar/gkl773 17062620 PMC1635334

[B18] Wang X Zhao Y Wong K Ehlers P Kohara Y Jones S Marra MA Holt RA Moerman DG Hansen D Identification of genes expressed in the hermaphrodite germ line of C. elegans using SAGE BMC Genomics 2009 10 213 10.1186/1471-2164-10-213 19426519 PMC2686737

[B19] Li SW Feng L Niu DK Selection for the miniaturization of highly expressed genes Biochem Biophy Res Commun 2007 360 3 586 592 10.1016/j.bbrc.2007.06.085 17610841

[B20] Stenoien HK Compact genes are highly expressed in the *moss Physcomitrella patens* J Evol Biol 2007 20 3 1223 1229 10.1111/j.1420-9101.2007.01301.x 17465932

[B21] Comeron JM What controls the length of noncoding DNA? Curr Opin Genet Dev 2001 11 652 659 10.1016/S0959-437X(00)00249-5 11682309

[B22] Petronv DA DNA loss and evolution of genome size in Drosophila Genetica 2002 115 81 91 10.1023/A:1016076215168 12188050

[B23] Castillo-Davis CI Hartl DL Achaz G Cis -regulatory and protein evolution in orthologous and duplicate genes Genome Res 2004 14 1530 1536 10.1101/gr.2662504 15256508 PMC509261

[B24] Chen J Sun M Hurst LD Carmichael GG Rowley JD Human antisense genes have unusually short introns: evidence for selection for rapid transcription Trends Genet 2005 21 4 203 207 10.1016/j.tig.2005.02.003 15797613

[B25] Jeffares DC Penkett CJ Bahler J Rapidly regulated genes are intron poor Trends Genet 2008 24 375 378 10.1016/j.tig.2008.05.006 18586348

[B26] Bradnam KR Korf I Longer first introns are a general property of eukaryotic gene structure PLoS One 2008 3 e3093 10.1371/journal.pone.0003093 18769727 PMC2518113

[B27] Haddrill P Charlesworth B Halligan D Andolfatto P Patterns of intron sequence evolution in Drosophila are dependent upon length and GC content Genome Biol 2005 6 R67 10.1186/gb-2005-6-8-r67 16086849 PMC1273634

[B28] Petit N Casillas S Ruiz A Barbadilla A Protein polymorphism is negatively correlated with conservation of intronic sequences and complexity of expression patterns in *Drosophila melanogaster* J Mol Evol 2007 64 511 518 10.1007/s00239-006-0047-5 17460807

[B29] Sironi M Menozzi G Comi GP Cagliani R Bresolin N Pozzoli U Analysis of intronic conserved elements indicates that functional complexity might represent a major source of negative selection on non-coding sequences Hum Mol Genet 2005 14 2533 2546 10.1093/hmg/ddi257 16037065

[B30] Versteeg R van Schaik BDC van Batenburg MF Roos M Monajemi R Caron H Bussemaker HJ van Kampen AHC The human Transcriptome Map Reveals Extremes in Gene Density, Intron Length, GC Content, and Repeat Pattern for Domains of Highly and Weakly Expressed Genes Genome Res 2003 13 1998 2004 10.1101/gr.1649303 12915492 PMC403669

[B31] Chamary JV Hurst LD Similar rates but different modes of sequence evolution in introns and at exonic silent sites in rodents: evidence for selectively driven codon usage Mol Biol Evol 2004 21 1014 1023 10.1093/molbev/msh087 15014158

[B32] Vinogradov AE Compactness of human housekeeping genes: selection for economy or genomic design? Trends Genet 2004 20 248 253 10.1016/j.tig.2004.03.006 15109779

[B33] Mourier T Jeffares DC Eukaryotic intron loss Science 2003 300 1393 10.1126/science.1080559 12775832

[B34] Niu DK Hou WR Li SW mRNA-mediated intron losses: evidence from extraordinarily large exons Mol Biol Evol 2005 22 1475 1481 10.1093/molbev/msi138 15788745

[B35] Camiolo S Rau D Porceddu A Mutational Biases and Selective Forces Shaping the Structure of Arabidopsis Genes PLoS ONE 2009 4 7 e6356 10.1371/journal.pone.0006356 19633720 PMC2712092

[B36] Huang YF Niu DK Evidence against the energetic cost hypothesis for the short introns in highly expressed genes BMC Evol Biol 2008 8 154 10.1186/1471-2148-8-154 18492248 PMC2424036

[B37] Singh J Padgett RA Rates of in situ transcription and splicing in large human genes Nat Struct Mol Biol 2009 16 1128 1133 10.1038/nsmb.1666 19820712 PMC2783620

[B38] Carmel L Rogozin IB Wolf YI Koonin EV Evolutionarily conserved gene preferentially accumulate introns Genome Res 2007 17 1045 1050 10.1101/gr.5978207 17495009 PMC1899115

[B39] Chan ET Quon GT Chua G Babak T Trochesset M Zirngibl RA Aubin J Ratcliffe M Wilde A Brudno M Morris OD Hughes TR Conservation of core gene expression in vertebrate tissues J Biol 2009 8 33 10.1186/jbiol130 19371447 PMC2689434

[B40] Zhang W Morris OD Chang R Shai O Bakowski MA Mitsakakis N Mohammad N Robinson MD Zirngibl R Somogyi E Laurin N Eftekharpour E Sat E Grigull J Pan Q Peng WT Krogan N Greenblatt J Fehlings M Kooy D Aubin J Bruneau BG Rossant J Blencowe BJ Frey BJ Hughes TR The functional landscape of mouse gene expression J Biol 2004 3 21 10.1186/jbiol16 15588312 PMC549719

[B41] Payseur BA Nachman MW Microsatellite Variation and Recombination Rate in the Human Genome Genetics 2000 156 1285 1298 11063702 10.1093/genetics/156.3.1285 PMC1461344

[B42] Beye M Gattermerer M Hasselmann I Gempe T Schioett M Exceptionally high levels of recombination across the honey bee genome Genome Res 2006 16 11 1339 44 10.1101/gr.5680406 17065604 PMC1626635

