# Supplementary material for: Selection for the compactness of highly expressed genes in Gallus gallus
Source: Biol Direct. 2010 May 14;5:35. doi: 10.1186/1745-6150-5-35 (PMC2883972; doi:10.1186/1745-6150-5-35)
Supplement: Additional file 2 — Scatter plots of CDS length, gene size, first intron length, average intron length versus gene expression level. (a) CDS length versus expression level (r = -0.4128, P = 0.01187); (b) gene length length versus expression level (r = -0.3122, P = 0.09273); (c) first intorn length versus expression level (r = -0.6679, P = 0.00683); (d) average intorn length versus expression level (r = -0.3978, P = 0.05106). Gene size, CDS length, first intron length, and average intron length are negatively correlated with expression level based on dataset 2. After adjusted values of type I error using a Bonferroni correction for multiple tests, the adjusted P- value for (a)and (c) remain statistically significantly, the adjusted P- value for (b) and (d) were 0.371 and 0.204, respectively. [file 1745-6150-5-35-S2.DOC]

(a) (b)

(c) (d)
